# Systemic Immune-Inflammation Index Is Associated With Increased Urinary Albumin Excretion: A Population-Based Study

**DOI:** 10.3389/fimmu.2022.863640

**Published:** 2022-03-21

**Authors:** Zheng Qin, Hancong Li, Liya Wang, Jiwen Geng, Qinbo Yang, Baihai Su, Ruoxi Liao

**Affiliations:** ^1^ Department of Nephrology, National Clinical Research Center for Geriatrics, West China Hospital, Sichuan University, Chengdu, China; ^2^ Med+ Biomaterial Institute of West China Hospital/West China School of Medicine of Sichuan University, Chengdu, China; ^3^ Med-X Center for Materials, Sichuan University, Chengdu, China; ^4^ West China School of Medicine, West China Hospital of Sichuan University, Chengdu, China

**Keywords:** systemic immune-inflammation index, albuminuria, population-based study, NHANES, cross-sectional study

## Abstract

**Background:**

Systemic immune-inflammation index (SII) is a novel inflammatory marker, and inflammation has been reported to be related with renal damage. We aimed to investigate the possible relationship between SII and albuminuria.

**Methods:**

The present cross-sectional study was conducted among adults with complete data about SII and urinary albumin-to-creatinine ratio (ACR) in 2005–2018 National Health and Nutrition Examination Survey (NHANES). SII was calculated as the platelet count × neutrophil count/lymphocyte count. Albuminuria was defined as ACR >30mg/g. Weighted multivariable regression analysis and subgroup analysis were conducted to explore the independent relationship between SII and albuminuria.

**Results:**

A total of 36,463 individuals were included in our analysis; 9.56% participants were categorized as having albuminuria overall and increased with the higher SII tertiles (tertile 1, 7.83%; tertile 2, 8.49%; tertile 3, 12.13%; p for trend <0.0001). Multivariable logistic regression showed that a higher SII level was associated with increased likelihood of albuminuria independently (OR = 1.31; 95% CI, 1.17–1.48, p<0.0001) after full adjustment. Subgroup analysis and interaction test showed that there was no significant dependence of gender, age, body mass index, hypertension, diabetes, non‐alcoholic fatty liver disease, and estimated glomerular filtration rate (eGFR) on this positive association (all p for interaction >0.05).

**Conclusions:**

SII was positively associated with increased urinary albumin excretion in US adults. Further large-scale prospective studies are still needed to analyze the role of SII in albuminuria.

## Introduction

Increased urinary albumin excretion is not only a marker of early kidney disease, but also has been shown to be an independent predictor of chronic kidney disease (CKD) progression and cardiovascular risk (1–4). Randomized urinary albumin-to-creatinine ratio (ACR) has been widely used as a method to assess and define albuminuria with great advantages of efficiency and convenience (5, 6). The recognized threshold for abnormal increase in ACR is 30 mg/g (3.4 mg/mmol). Microalbuminuria (30–300 mg/g) is reported to occur in 5–19% of the general population, up to 23% in hypertensive patients, and up to 40% in diabetic patients (7). Due to its high incidence and significant negative impact on unfavorable clinical outcomes, albuminuria has emerged as a major public health problem (8–10). Consequently, high clinical attention should be paid to albuminuria.

Systemic immune-inflammation index (SII) has been considered as a good, stable index, which could reflect the local immune response and systemic inflammation in the whole human body (11–13). It integrated three types of inflammatory cells, including platelet, neutrophil, and lymphocyte, and was calculated by platelet count × neutrophil count/lymphocyte count, which was first developed by Hu et al. in 2014 and has been investigated widely (14). Several studies confirmed its high prognostic values in several tumors, such as colorectal cancer (11), cervical cancer (13), hepatocellular cancer (9), lung cancer (15), esophageal cancer (16), and epithelial ovarian cancer (17). To our interest, SII may also be associated with adverse outcomes for other malignant diseases in addition to tumors. Yang et al. reported that SII showed a better predictive value of major cardiovascular events than traditional risk factors in coronary artery disease patients after coronary intervention (18). In addition, SII was also proved to be an independent risk factor for protein energy wasting in maintenance hemodialysis patients (19).

Many studies have shown that inflammation plays a role in the decline in kidney function. A population-based cohort study evaluating clinical and biological determinants of renal function found that C-reactive protein (CRP) level was associated with adverse kidney outcomes including rapid decline in estimated glomerular filtration rate (eGFR) and incidence of CKD (20). Shankar et al. reported that inflammatory biomarkers (CRP, TNF-αR2, white blood cell count, and IL-6) were positively associated with the outcome of prevalent CKD (21). A recent review referred to an intermediate phenotype involving chronic inflammation, oxidative stress, hypoxia, and mitochondrial dysfunction, which play key roles in the etiology, pathophysiology. and progression of CKD (22). Inflammation has been proven to be related with renal damage; however, the relationship between inflammatory level indicator SII and albuminuria has not been clearly defined.

Therefore, the aim of our study was to explore the association between SII and albuminuria among the participants of the US National Health and Nutrition Examination Survey (NHANES). We assumed that an elevated SII would be associated with a higher risk of albuminuria.

## Subjects and Methods

### Data and Sample Sources

Data were obtained from NHANES, a national population-based cross-sectional survey to collect the information about the potential health risk factors and nutrition status of US non-institutionalized civilian, which was conducted by the National Center for Health Statistics (NCHS). A complex stratified, multistage probability cluster sampling design were designed to recruit a representative sample of the whole US population (23). The protocols for the NHANES study were approved by The Research Ethics Review Board of the NCHS. Written informed consent was obtained from all survey participants or from a parent and/or legal guardian for participants below 16 years old. The detailed NHANES study design and data are publicly available at https://www.cdc.gov/nchs/nhanes/.

Participants received a standardized in-home interview and health examination at mobile examination centers to assess their medical and physiological status, and laboratory tests were conducted to collect their laboratory data. Seven NHANES cycles from 2005 to 2018 were selected to assess the association between SII and increased urinary albumin excretion, since only these survey cycles included complete variables to calculate SII, ACR, and eGFR using the same protocols.

**Figure 1:**
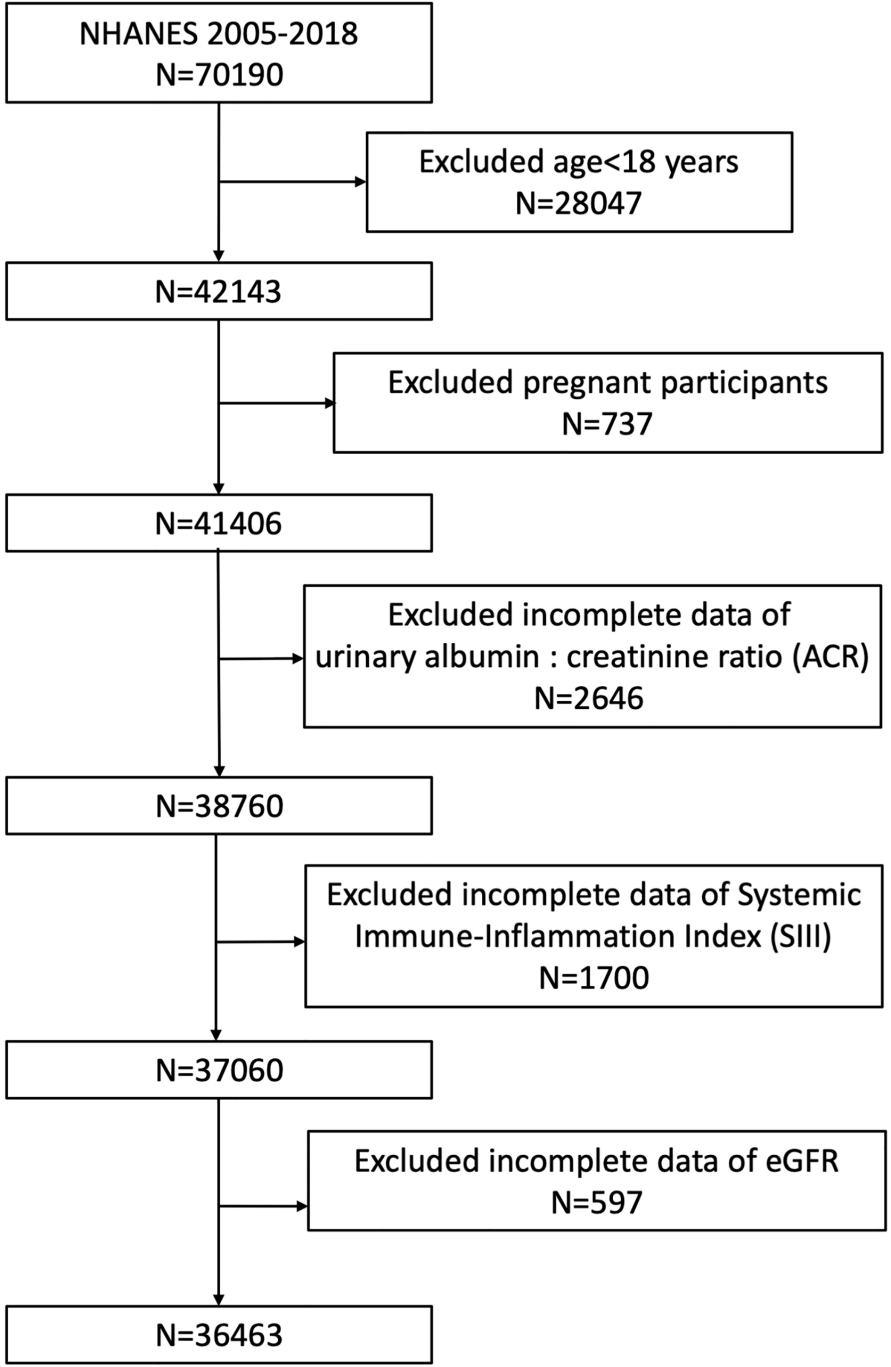
Flowchart of the sample selection from NHANES 2005–2018.

The exclusion criteria for participants in our analysis were (1) aged <18 years old, (2) pregnant, and (3) missing complete data about ACR, SII, and eGFR. A total of 70,190 participants was enrolled at first; after the exclusion of participants aged <18 years (n=28,047), who were pregnant (n=737), and with missing data about ACR (n=2,646), SII (=1,700), and eGFR (n=597), 36,463 eligible participants aged ≥18 years were included in our final analysis (**Figure 1**).

### Assessment of Increased Urinary Albumin Excretion

Blood and urine samples of NHANES participants were obtained at a standardized mobile examination center. Urinary albumin and creatinine were determined by a solid-phase fluorescent immunoassay and modified Jaffe kinetic method using a single, spot urine sample. ACR was calculated by dividing the urinary albumin concentration in milligrams by the urinary creatinine concentration in grams. Increased urinary albumin excretion (albuminuria) was defined as ACR >30 mg/g (24, 25). Albuminuria was treated as an outcome variable in our analysis.

### Definition of Systemic Immune-Inflammation Index

Lymphocyte, neutrophil, and plate counts were measured by complete blood count using automated hematology analyzing devices (Coulter^®^ DxH 800 analyzer) and present as ×10^3^ cells/μl. We calculated SII as plate count × neutrophil count/lymphocyte count as previous studies reported (14, 26). SII was designed as exposure variable in our analysis.

### Covariates

Covariates that may affect the association between SII and albuminuria were included in our study as well, including gender (male/female), age (year), race (Mexican American/other Hispanic/non-Hispanic White/non-Hispanic Black/other races), education level (less than high school/high school or general educational development/above high school/others), smoking status (never/former/current/unknown), physical activity (vigorous/moderate/less than moderate), body mass index (BMI, kg/m^2^), systolic blood pressure (SBP, mmHg), diastolic blood pressure (DBP, mmHg), hypertension (yes/no), diabetes (yes/no), non‐alcoholic fatty liver disease (NAFLD, yes/no), fasting plasma glucose (uIU/ml), serum creatinine (SCr, mg/dl), serum uric acid (μmol/L), total cholesterol (mmol/L), high-density lipoprotein-cholesterol (HDL-C), low-density lipoprotein-cholesterol (LDL-C), alanine transaminase (ALT, IU/L), aspartate transaminase (AST, IU/L), triglycerides (mmol/L), waist circumference (cm), and estimated-glomerular filtration rate (eGFR, ml/min/1.73m^2^). Serum creatinine (SCr) was determined by Jaffe rate method and calibrated by standardized isotope dilution mass spectrometry. Data about gender, race, age, and SCr were used to calculate estimated glomerular filtration rate (eGFR) according to the CKD Epidemiology Collaboration (CKD-EPI) creatinine equation for each participants (27). eGFR was categorized as <60, 60–89.9, and ≥90 ml/min/1.73m^2^ in subgroup analysis. BMI was categorized as <25, 25–29.9 and ≥30 kg/m^2^, which corresponded to normal weight, overweight, and obese population for participants. Serum ALT concentrations were detected either using an enzymatic rate method with Beckman Synchron LX20 (NHANES 2005-2006) or using a kinetic rate method with Beckman UniCel DxC800 Synchron (NHANES 2007-2018). Suspect NAFLD individuals were ascertained based on serum ALT, which was commonly used as a screening test and monitoring biomarker for NAFLD (28, 29). Based on previous studies, NAFLD status was assumed as serum ALT >30 IU/L in men and >19 IU/L in women with the absence of significant alcohol consumption (>3 drinks/day in men and >2 drinks/day in women) and/or viral hepatitis (hepatitis B virus or hepatitis C virus infections) in our analysis (30, 31). All detailed measurement processes of these variable were publicly available at www.cdc.gov/nchs/nhanes/.

### Statistical Analysis

All statistical analysis was conducted according to the Centers for Disease Control and Prevention (CDC) guidelines using an appropriate NHANES sampling weights and accounted for complex multistage cluster survey. Continuous variables were summarized as means with standard error (SE), and categorical parameters were presented as proportions. Either a weighted Student’s t-test (for continuous variables) or weighted chi-square test (for categorical variables) was employed to evaluate the differences among participants grouped by SII tertiles. Multivariable logistic regression was used to test the association between SII and albuminuria in three different models. In model 1, no covariates were adjusted. In model 2, gender, age, and race were adjusted. Model 3 was adjusted for gender, age, race, education level, physical activity, ALT, AST, total cholesterol, SCr, triglycerides, serum uric acid, BMI, waist circumference, SBP, DBP, fasting plasma glucose, HDL-C, LDL-C, eGFR, hypertension, diabetes, NAFLD, and smoking status. It was noted that SII was log2-transformed when conducting regression analysis because they were right-skewed distributed. Subgroups analysis on the associations of SII with albuminuria was conducted with stratified factors including gender (male/female), age (<60/≥ 60 years), BMI (normal weight/overweight/obesity), hypertension (yes/no), diabetes (yes/no), NAFLD (yes/no), and (eGFR, <60/60–89.9/≥90 ml/min/1.73 m^2^). These stratified factors were also treated as pre-specified potential effect modifiers. An interaction term was added to test the heterogeneity of associations between the subgroups as well. Missing values were input by median for continuous variables or mode for categorical variables of existing cases of those variables. All analyses were performed using R version 3.4.3 (http://www.R-project.org, The R Foundation) and Empower software (www.empowerstats.com; X&Y solutions, Inc., Boston, MA). The statistically significance level was set as p<0.05.

## Results

### Baseline Characteristics of Participants

A total of 36,463 participants were enrolled, of whom 49.04% were male and 50.96% were female, with an average age of 46.65 ± 0.23 years; 9.56% participants were categorized as having increased urinary albumin excretion overall and increased with the higher SII tertiles. The prevalence of albuminuria was 7.83%, 8.49%, and 12.13% in tertile 1, tertile 2 and tertile 3, respectively. Among three SII tertiles, differences with statistical significance were observed in age, gender, race, smoking status, physical activity, BMI, SBP, DBP, diabetes, hypertension, SCr, total cholesterol, HDL-C, ALT, AST, waist circumference, triglycerides, eGFR, urinary albumin, and ACR (all p<0.05). Subjects with increased SII level were female, smoker, had elevated age, BMI, SBP, diabetes, hypertension, fasting plasma glucose, triglycerides, waist circumference, and urinary albumin, and decreased HDL-C, ALT, and eGFR levels in our study (all p<0.05). The clinical and biochemical characteristics of the participants according to SII quartiles are shown in **Table 1**.

**Table 1:** Baseline characteristics of study population according to systemic immune-inflammation index tertiles, weighted.

Systemic Immune-Inflammation Index	Overall	Tertile 1	Tertile 2	Tertile 3	p for trend
Age (year)	46.65 ± 0.23	45.64 ± 0.31	46.43 ± 0.24	47.76 ± 0.30	<0.0001
Gender, % (SE)					
Male	49.04 (0.27)	54.60 (0.66)	49.37 (0.51)	43.82 (0.57)	<0.0001
Female	50.96 (0.27)	45.40 (0.66)	50.63 (0.51)	56.18 (0.57)	
Race, % (SE)					
Mexican American	8.75 (0.68)	8.84 (0.70)	8.89 (0.72)	8.55 (0.70)	<0.0001
Other Hispanic	5.62 (0.44)	5.76 (0.44)	5.75 (0.48)	5.39 (0.49)	
Non-Hispanic White	67.15 (1.28)	59.79 (1.54)	68.68 (1.33)	72.09 (1.21)	
Non-Hispanic Black	10.75 (0.67)	16.77 (1.03)	8.91 (0.57)	7.31 (0.52)	
Other Races	7.72 (0.39)	8.84 (0.54)	7.78 (0.48)	6.67 (0.40)	
Education level, % (SE)					
Less than high school	16.50 (0.55)	17.06 (0.66)	15.97 (0.61)	16.52 (0.64)	0.2469
High school or GED	23.67 (0.47)	22.65 (0.69)	23.21 (0.62)	25.03 (0.58)	
Above high school	59.77 (0.82)	60.23 (1.04)	60.72 (0.88)	58.42 (0.96)	
Others	0.06 (0.01)	0.05 (0.03)	0.09 (0.03)	0.03 (0.01)	
Smoking status, % (SE)					
Never	54.41 (0.53)	56.87 (0.68)	55.93 (0.67)	50.75 (0.78)	<0.0001
Former	23.97 (0.40)	23.76 (0.57)	23.19 (0.47)	24.95 (0.60)	
Current	19.76 (0.42)	17.08 (0.56)	19.27 (0.55)	22.60 (0.54)	
Unknown	1.85 (0.09)	2.29 (0.17)	1.61 (0.13)	1.71 (0.13)	
Physical activity, % (SE)					
Vigorous	38.70 (0.54)	42.93 (0.91)	39.77 (0.67)	33.54 (0.72)	0.0021
Moderate	29.64 (0.47)	28.26 (0.77)	29.53 (0.73)	31.08 (0.65)	
Less than moderate	31.66 (0.49)	28.81 (0.70)	30.70 (0.73)	35.37 (0.64)	
BMI (kg/m^2^)	28.90 ± 0.08	27.98 ± 0.10	28.83 ± 0.11	29.77 ± 0.11	<0.0001
SBP (mmHg)	122.40 ± 0.18	121.60 ± 0.25	122.17 ± 0.24	123.34 ± 0.23	<0.0001
DBP (mmHg)	70.70 ± 0.18	70.28± 0.22	70.99 ± 0.20	70.77± 0.22	0.0236
Diabetes, % (SE)	9.24 (0.23)	8.12 (0.36)	8.78 (0.30)	10.67 (0.40)	<0.0001
Hypertension, % (SE)	30.94 (0.47)	28.30 (0.71)	29.58 (0.62)	34.60 (0.68)	<0.0001
NAFLD, % (SE)	35.80 (0.36)	35.68 (0.59)	36.34 (0.56)	35.36 (0.60)	0.6321
Fasting plasma glucose (uIU/ml)	5.92 ± 0.02	5.82 ± 0.03	5.92 ± 0.03	6.02 ± 0.03	<0.0001
Serum creatinine (μmol/L)	78.39 ± 0.22	79.21 ± 0.26	77.85 ± 0.31	78.20 ± 0.37	0.0349
Serum uric acid (μmol/L)	322.56 ± 0.73	322.29 ± 1.08	321.76 ± 1.12	323.61 ± 1.10	0.3535
Total cholesterol (mmol/L)	5.00 ± 0.01	4.94 ± 0.02	5.04 ± 0.02	5.02 ± 0.02	0.0004
HDL-C (mmol/L)	1.38 ± 0.01	1.40 ± 0.01	1.37 ± 0.01	1.38 ± 0.01	0.0174
LDL-C (mmol/L)	2.93 ± 0.01	2.89 ± 0.02	2.99 ± 0.02	2.92 ± 0.02	0.1904
ALT (IU/L)	25.22 ± 0.13	25.97 ± 0.26	25.38 ± 0.20	24.41 ± 0.21	<0.0001
AST (IU/L)	25.23 ± 0.10	26.45 ± 0.21	24.77 ± 0.13	24.62 ± 0.17	<0.0001
Triglycerides (mmol/L)	1.71 ± 0.01	1.62 ± 0.02	1.74 ± 0.02	1.75 ± 0.02	<0.0001
Waist circumference (cm)	98.70 ± 0.21	96.35 ± 0.28	98.68 ± 0.26	100.80 ± 0.26	<0.0001
eGFR (ml/min/1.73 m^2^)	94.61 ± 0.31	96.04 ± 0.36	94.82 ± 0.37	93.14 ± 0.42	<0.0001
Albumin, urine (mg/L)	33.83 ± 1.32	25.84 ± 1.50	29.69 ± 1.77	44.93 ± 3.03	<0.0001
Creatinine, urine (mg/dl)	122.22 ± 0.83	124.32 ± 1.27	120.95 ± 1.16	121.64 ± 1.07	0.0902
ACR (mg/g)	33.38 ± 1.38	24.16 ± 1.55	28.59 ± 1.96	46.23 ± 3.10	<0.0001
Albuminuria, % (SE)	9.56 (0.22)	7.83 (0.31)	8.49 (0.32)	12.13 (0.42)	<0.0001

GED, general educational development; BMI, body mass index; SBP, systolic blood pressure; DBP, diastolic blood pressure; NAFLD, non‐alcoholic fatty liver disease; HDL-C, high-density lipoprotein-cholesterol; LDL-C, low-density lipoprotein-cholesterol; ALT, alanine transaminase; AST, aspartate transaminase; ACR, albumin:creatinine ratio.

### The Association Between SII and Increased Urinary Albumin Excretion

Our results showed that higher SII was associated with increased likelihood of increased urinary albumin excretion. This association was significant both in our crude model (OR=1.39; 95% CI, 1.31–1.47, p<0.0001) and minimally adjusted model (OR=1.39; 95% CI, 1.32–1.47, p<0.0001). In the fully adjusted model, the positive association between SII and albuminuria still remained stable (OR = 1.31; 95% CI, 1.17–1.48, p<0.0001), indicating that each unit of increased log2-SII score was associated with 31% increased risk of increased urinary albumin excretion, respectively. We further converted SII from a continuous variable to a categorical variable (tertiles) to conduct the sensitivity analysis. Compared with the lowest SII tertile, participants in the highest SII tertile had significantly 50% increased risk of albuminuria than those in the lowest SII tertile with statistical significance (OR=1.50; 95% CI, 1.21–1.87, p=0.0005). Participants in the middle SII tertile also show a higher risk of albuminuria compared with the lowest tertile, while this association did not meet the statistical significance (OR=1.13; 95% CI, 0.92–1.38, P=0.2457) (**Table 2**).

**Table 2:** Association Between Systemic Immune-Inflammation Index and Albuminuria.

	OR^1^ (95%CI^2^), p-value		
	Crude model	Minimally adjusted model	Fully adjusted model
	(Model 1)^3^	(Model 2)^4^	(Model 3)^5^
Continuous			
	1.39 (1.31, 1.47),	1.39 (1.32, 1.47),	1.31 (1.17, 1.48),
	<0.0001	<0.0001	<0.0001
Categories			
Tertile 1	Reference	Reference	Reference
Tertile 2	1.09 (0.97, 1.23),	1.14 (1.01, 1.29),	1.13 (0.92, 1.38),
	0.1564	0.0355	0.2457
Tertile 3	1.63 (1.46, 1.80),	1.67 (1.50, 1.86),	1.50 (1.21, 1.87),
	<0.0001	<0.0001	0.0005
P for trend	<0.0001	<0.0001	<0.0001

In sensitivity analysis, SII was converted from a continuous variable to a categorical variable (tertiles).

^1^OR: odds ratio.

^2^95% CI: 95% confidence interval.

^3^Model 1: no covariates were adjusted.

^4^Model 2: adjusted for gender, age, and race.

^5^Model 3: adjusted for gender, age, race, education level, physical activity, ALT, AST, total cholesterol, serum creatinine, triglycerides, serum uric acid, body mass index, waist circumference, systolic blood pressure, diastolic blood pressure, fasting plasma glucose, HDL-C, LDL-C, eGFR, hypertension, diabetes, NAFLD, and smoking status.

Age, gender, race, SBP, diabetes, hypertension, fasting plasma glucose, SCr, serum uric acid, and eGFR remained significantly associated with the odds of having albuminuria in the fully adjusted model (**Table 3**). Compared with male participants, female participants had 1.6 times higher odds of albuminuria (P<0.0001). Compared with Mexican American, other Hispanic, non-Hispanic White, and non-Hispanic Black had 25%, 33%, and 52% lower likelihood of albuminuria (all p<0.0001). The odds of albuminuria were 46% and 30% in non-diabetes and non-hypertension compared with diabetes (p<0.0001) and hypertension (p=0.0002). Per unit increase in fasting plasma glucose, SCr, serum uric acid, and eGFR, the odds of albuminuria were elevated by 12% (p<0.0001), 4% (p<0.0001), 0.2% (p<0.0001) and 4% (p<0.0001), respectively.

**Table 3:** Multivariate logistic regression models of albuminuria.

Variables	OR^1^ (95% CI^2^)	p-value
Systemic Immune-Inflammation Index	1.31 (1.17, 1.48)	<0.0001
Age (year)	1.04 (1.02, 1.05)	<0.0001
Female (versus male)	2.60 (1.83, 3.70)	<0.0001
Race (versus Mexican American)		
Other Hispanic	0.75 (0.56, 0.99)	0.0464
Non-Hispanic White	0.67 (0.54, 0.83)	0.0005
Non-Hispanic Black	0.48 (0.36, 0.64)	<0.0001
Other Races	0.90 (0.68, 1.20)	0.4791
Education level (versus less than high school)		
High school or GED	0.96 (0.78, 1.17)	0.6730
Above high school	0.74 (0.57, 0.97)	0.0301
Others	0.67 (0.13, 3.58)	0.6447
Smoke (versus never)		
Former	1.11 (0.89, 1.38)	0.3754
Current	1.35 (1.04, 1.75)	0.0273
Unknown	1.29 (0.68, 2.43)	0.4396
Physical activity (versus vigorous)		
Moderate	1.02 (0.79, 1.32)	0.8844
Less than moderate	1.31 (0.98, 1.75)	0.0673
BMI (kg/m^2^)	1.01 (0.97, 1.04)	0.6976
SBP (mmHg)	1.02 (1.01, 1.03)	<0.0001
DBP (mmHg)	1.002 (0.995, 1.009)	0.5649
Diabetes (no versus yes)	0.54 (0.43, 0.68)	<0.0001
Hypertension (no versus yes)	0.70 (0.59, 0.84)	0.0002
NAFLD (yes versus no)	0.85 (0.69, 1.05)	0.1285
Fasting plasma glucose (uIU/ml)	1.12 (1.08, 1.16)	<0.0001
Serum creatinine (μmol/L)	1.04 (1.03, 1.05)	<0.0001
Serum uric acid (μmol/L)	1.002 (1.000, 1.003)	0.0123
Total cholesterol (mmol/L)	1.30 (0.84, 2.01)	0.2451
HDL-C (mmol/L)	0.96 (0.59, 1.56)	0.8639
LDL-C (mmol/L)	0.72 (0.47, 1.12)	0.1538
eGFR (ml/min/1.73 m^2^)	1.04 (1.02, 1.06)	<0.0001
ALT (IU/L)	0.996 (0.986, 1.006)	0.4412
AST (IU/L)	1.01 (1.00, 1.02)	0.1303
Triglycerides (mmol/L)	1.01 (0.81, 1.25)	0.9475
Waist circumference (cm)	0.997 (0.983, 1.012)	0.7266

^1^OR: odds ratio.

^2^95% CI: 95% confidence interval.

The unit for continuous variables and the reference group for categorical variables are provided next to the variables. The OR of albuminuria was each unit increase in continuous variables and compared with the reference group for categorical variables.

### Subgroup Analysis

**Figure 2:**
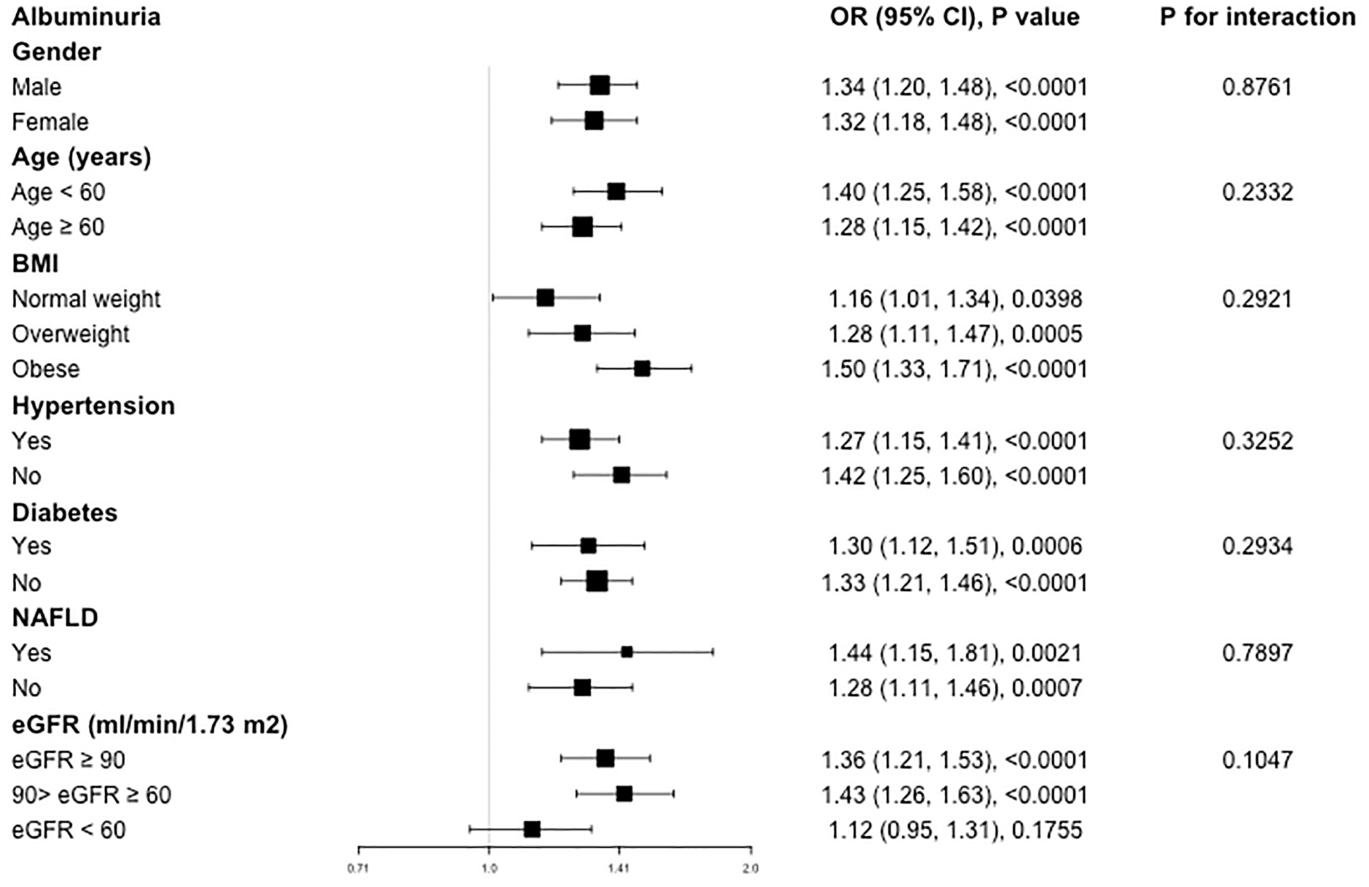
Subgroup analysis for the association between SII and albuminuria.

Our results of subgroup analysis indicated that the associations of SII level with increased urinary albumin excretion was not consistently the same (**Figure 2**). For the subgroup stratified by gender, age, BMI, hypertension, diabetes, and NAFLD, a significant relationship of SII with albuminuria was detected in each subgroup (all p<0.05). As for the subgroup stratified by eGFR, association with statistical significance was only observed in those with eGFR ≥60 ml/min/1.73m^2^ (p<0.05). For participants with eGFR <60 ml/min/1.73m^2^, a positive association between SII and albuminuria was also observed, while this association did not meet the statistical significance (OR=1.12; 95%CI, 0.95–1.31, p=0.1755). The interaction test showed that there was no significant difference among each stratification in the association between SII and albuminuria, indicating that there was no significant dependence of gender, age, BMI, hypertension, diabetes, NAFLD and eGFR on this positive association (all p for interaction >0.05).

## Discussion

In our cross-sectional study with 36,463 participants enrolled, we observed that participants with higher SII showed increased likelihood of albuminuria. Subgroup analysis and interaction test showed that this association was similar in different population settings. Our finding suggested that higher SII levels were an independent risk factor for increased urinary albumin excretion.

To our knowledge, this is the first study assessing the association between SII and increased urinary albumin excretion. Previous studies have reported the association between SII and kidney diseases with varying epidemiological methods and target populations (32–34). A population-based cohort with 218 severe acute pancreatitis (SAP) patients in China suggested that SII was a simple and powerful marker for early prediction of acute kidney injury (AKI) in SAP patients with great accuracy (32). To be specific, the optimal SII cutoff point was 2,880.1×10^9^, and SII had a better predictive effect compared with other inflammatory factors such as CRP, triglyceride, and serum albumin (32). Similarly, qualification of SII as a novel independent predictor of postoperative AKI in hepatocellular carcinoma patients has been reported as well (33). Another retrospective study found the association between SII and increased TNM stage and poor prognosis of renal cell carcinoma patients undergoing radical nephrectomy (34). However, there have also been several studies that revealed the limitations of SII (35, 36). A study that enrolled 378 patients who underwent primary kidney transplantation (KTx) in America reported that SII demonstrated limited utility as an independent predictor of outcomes after KTx, while in combination with other clinically relevant parameters, SII could be a useful predictor of prognosis after KTx (35). Another meta-analysis with 2,693 subjects included suggested that elevated SII indicated poor prognosis in patients with urinary system cancers. Nevertheless, given the substantial heterogeneity and limited number of studies included, future large-scale and multi-center studies are still needed to verify the findings (36). Wijarnpreecha et al. reported that patients with NAFLD had a significantly increased risk of albuminuria compared with non-NAFLD (37). Similarly, our subgroup analysis demonstrated that for each increase in log2 SII, NAFLD participants showed a higher risk of albuminuria than those without NAFLD (NAFLD: OR=1.44; 95%CI, 1.15–1.81; p=0.0021; non-NAFLD: OR=1.28; 95%CI, 1.11–1.46, p=0.0007), indicating that more attention should be paid for individuals with albuminuria especially in patients with NAFLD. Consistent with most studies, our study demonstrated that a higher SII was independently associated with increased risk of albuminuria, suggesting that SII may have a significant negative impact on renal function independently.

Although SII has been regarded as a novel and available inflammatory marker, there are many classic inflammatory indicators in clinical practice with widely clinical applications. A population-based study of 4,926 patients from Wisconsin found that markers of inflammation, including high-sensitivity CRP, TNF-α R2, WBC count, and IL-6 levels, were related to prevalent CKD in the cross-sectional analysis (21). Previous studies also reported that IL-6 was elevated in most end-stage renal disease (ESRD) patients and may play a central role in the pathophysiology of inflammation in patients with ESRD, which was related with mortality (38, 39). In addition, epidemiological studies indicated that increased CRP was a strong predictor of morbidity and mortality in ESRD patients (40–42). A recent population-based cohort study also demonstrated that CRP (OR=1.16; 95% CI, 1.05–1.22) was associated with rapid decline in eGFR or incident CKD (20). Furthermore, several animal studies also suggested that inflammation may bring about decreased renal function and CKD development (43–45). Tomosugi et al. reported that rats pretreated with TNF-α and IL-6 showed increasing severity of glomerular injury (43). Transgenic mice that constitutively express the IL-6 in the liver and secrete it into the blood could develop a progressive kidney disease, resulting in serum protein overload in the kidney damage (44). Another study documented dose-dependent damage to glomerular endothelial cells in rabbits injected with increased doses of human recombinant TNF-α (45). In addition to positive inflammatory marker, such as CRP, ferritin, or fibrinogen, which are usually elevated during an acute episode of inflammation, there has also been a documented decrease in serum levels of negative acute phase reactants, such as albumin or transferrin during inflammation (46, 47). Compared to traditional inflammatory factors, SII reflected the inflammatory state preferably and has shown better prognostic value in several studies (11–14, 32, 48). For example, the ability of the SII, neutrophil/lymphocyte ratio (NLR), platelet/lymphocyte ratio (PLR), and monocyte/lymphocyte ratio (MLR) to predict postoperative survival of patients with cervical cancer was studied by Huang et al. Their ROC curve analysis confirmed that, among these markers, the SII was more effective and accurate in predicting the outcomes of patients with cervical cancer (13). Similarly, such better predictive performance compared with other inflammatory factors was also reported in early prediction of AKI in SAP patients (32). SII also has been confirmed to be more promising than NLR or PLR in several clinical settings (11, 12, 14, 48).To sum up, SII has shown outstanding predictive power in a variety of studies, and simultaneously, SII was a universally available method characterized by a non-invasive approach, easy access, and low cost. It thus has promising prospects for clinical application.

The exact mechanism of the positive association between inflammation and decreased renal function still remains unclear. Elevated TNF-α could lead to glomerulosclerosis and decreased renal function in patients with CKD by enhancing glomerular oxidative stress. In addition, TNF-α may also lead to glomerular damage and proteinuria by increasing glomerular mononuclear cell infiltration, thus accelerating the progression of CKD (49). Viecelli et al. suggested that the interaction between inflammatory mediators could cause vein stenosis near the graft-vein anastomosis site, leading to vascular access failure, which may be one of the underlying mechanisms (50).

Our study holds its own strengths. First, sample selection is representative, and the sample size is large enough. Second, we adjusted for confounding factors to produce more reliable results. For example, previous studies reported that physical activity may improve the endothelial function to reduce albuminuria, and regular physical activity was associated with lower inflammatory markers (51, 52). Thus, we treated physical activity as a covariate in our fully adjusted model to make our analysis more reliable. However, the results of this study should be interpreted with caution for several limitations. First, due to the cross-sectional study design, we could not obtain a causal relationship. Therefore, prospective studies with larger sample size are still required to clarify the causality. Additionally, although we adjusted some potential covariates, we could not completely exclude the influence of other possible confounding factors.

## Conclusion

Our study demonstrated that elevated SII level was associated with increased urinary albuminuria excretion independently. Further large-scale prospective studies are still needed to validate our findings.

## Data Availability Statement

Publicly available datasets were analyzed in this study. These data can be found here: https://www.cdc.gov/nchs/nhanes/.

## Ethics Statement

The studies involving human participants were reviewed and approved by the Research Ethics Review Board of the NCHS. Written informed consent to participate in this study was provided by the participants’ legal guardian/next of kin.

## Author Contributions

ZQ: data analysis and writing—original draft; HL: writing—original draft; LW: software; JG: methodology; QY: writing—original draft; BS: methodology, conceptualization, and funding acquisition; RL: conceptualization and writing—reviewing and editing. All authors contributed to the article and approved the submitted version.

## Funding

This work was supported by the National Natural Science Foundation of China (Grant No. 82000702), the Science and Technology Achievement Transformation Fund of West China Hospital of Sichuan University (Grant No. CGZH19006), the 1.3.5 project for disciplines of excellence from West China Hospital of Sichuan University (Grant No. ZYJC21010), National Clinical Research Center for Geriatrics, West China Hospital, Sichuan University (Grant No. Z2018B10) and Med+ Biomaterial Institute of West China Hospital/West China School of Medicine of Sichuan University (Grant No. ZYME20001).

## Conflict of Interest

The authors declare that the research was conducted in the absence of any commercial or financial relationships that could be construed as a potential conflict of interest.

## Publisher’s Note

All claims expressed in this article are solely those of the authors and do not necessarily represent those of their affiliated organizations, or those of the publisher, the editors and the reviewers. Any product that may be evaluated in this article, or claim that may be made by its manufacturer, is not guaranteed or endorsed by the publisher.
